# Translation and validation of the Chinese version of the Japan Frailty Scale

**DOI:** 10.3389/fmed.2023.1257223

**Published:** 2023-09-27

**Authors:** Dongping Wan, Rui Wang, Jie Wei, Qiang Zan, Lei Shang, Jianbing Ma, Shuxin Yao, Chao Xu

**Affiliations:** ^1^Department of Knee Joint Surgery, Honghui Hospital, Xi’an Jiaotong University, Xi’an, China; ^2^The First Clinical Medical College, Shaanxi University of Chinese Medicine, Xianyang, China; ^3^State Key Laboratory of Cancer Biology, Department of Pathology, The First Affiliated Hospital of Air Force Military Medical University, Xi’an, China; ^4^Department of Health Statistics, Faculty of Preventive Medicine, The Air Force Military Medical University, Xi’an, China

**Keywords:** the Japan Frailty Scale, Chinese version, reliability, validity, culturally adaption

## Abstract

**Purpose:**

Frailty is a difficult-to-measure condition that is susceptible to adverse outcomes. The Japan Frailty Scale (JFS) is a tool for assessing frailty status in older adults. This study aimed to translate and culturally adapt the JFS into a Chinese version (JFS-C).

**Materials and methods:**

The study included 160 older adults as participants. Internal consistency was assessed using Cronbach’s alpha, and test–retest reliability was conducted using the intraclass correlation coefficient (ICC). Convergent validity was evaluated by assessing the correlation between JFS-C and the Barthel Index, the Frail scale, and the 36-item Short-Form Health Survey (SF-36). Criterion validity was assessed by comparing JFS-C scores with the Frail scale.

**Results:**

JFS-C demonstrated adequate internal consistency (Cronbach’s alphas = 0.711) and excellent test–retest reliability over a 7 to 10-day interval (ICC = 0.949). Correlation analysis showed a strong positive correlation between JFS-C and the Frail scale (*r* = 0.786, *p* < 0.001), a moderate negative correlation with the Barthel Index (*r* = −0.598, *p* < 0.001), and moderate correlations with various subscales of SF-36 (*r* = −0.574 to −0.661). However, no significant correlations were found between JFS-C and SF-36 mental health (*r* = −0.363, *p* < 0.001) or role emotional (*r* = −0.350, *p* < 0.001). Based on the reference standard of the Frail scale phenotype (score ≥ 2), the cutoff value for JFS-C was determined to be 3.

**Conclusion:**

JFS-C demonstrates good reliability and validity in assessing frailty among the older population in China.

## 1. Introduction

Frailty, a prevalent geriatric syndrome, is characterized by diminished physiological reserves and functional capacity, resulting in heightened vulnerability to adverse health outcomes (1, 2). In older adults, frailty is associated with an increased risk of falls, hospitalization, disability, and mortality (3). Studies have reported a wide range of frailty prevalence among older adults residing in Chinese communities, ranging from 4.0% to 59.1% (4), underscoring significant health concerns within the general population. Consequently, the identification of frailty status plays a crucial role in developing effective intervention strategies aimed at preventing or delaying adverse outcomes in the older population.

Frailty presents a challenge due to its lack of a precise conceptual definition and necessitates the use of diverse objective or subjective measures. Objective assessments, including metrics such as gait speed, grip strength, and muscle mass (5, 6), have proven to be predictive of adverse outcomes in older individuals. These indicators are frequently combined with subjective scales to evaluate the severity of frailty (7). Subjective tools, such as questionnaires and interviews, offer a convenient and cost-effective means of assessing frailty across multiple domains, including the physical, psychological, and social aspects (8). These tools can be easily administered in various healthcare settings, such as community health clinics and primary care offices (9, 10). By incorporating subjective scales, a more comprehensive assessment of frailty is achieved, capturing the psychosocial factors that contribute to frailty. This comprehensive evaluation facilitates effective communication between healthcare providers and older adults, ensuring that specific areas of concern are identified and addressed.

Currently, there are several tools available for frailty assessment. The Frailty Phenotype (FP) measures five physiological indicators, omitting considerations for cognitive and psychosocial aspects, and necessitating specialized equipment such as dynamometers for assistance (11), thus limiting its clinical applicability. In contrast, The Frailty Index (FI) compiles accumulated deficits encompassing physical performance, cognitive function, and psychosocial aspects, with researchers able to customize their FI through standardized procedures (12); however, a consistent criterion for identifying potential variables linked to the frailty index is still under development (13). While the FRAIL scale incorporates outcomes from five domains, four of its constituent components conceptually overlap with the CHS index (14), resulting in a lack of originality.

The Japan Frailty Scale (JFS) is a patient-reported screening tool specifically designed within the context of Kampo medicine’s aging concept to evaluate frailty (15). Extensive research has demonstrated the JFS’s robust reliability and validity in Japanese populations (15). Kampo medicine originated in China and is based on traditional Chinese medicine (16), adapted to Japanese culture (17). In addition, both traditional Chinese medicine and Kampo medicine are rooted in philosophical theories from East Asian traditions, such as the Five Elements theory that incorporates the principles of Yin and Yang (18). Both traditional Chinese medicine and Kampo medicine uphold the importance of the “Yellow Emperor’s Classic of Internal Medicine” (19). The items (symptoms) in the JFS questionnaire are selected from the traditional Chinese text “Yellow Emperor’s Classic of Internal Medicine” (15). In China, a validated scale for assessing frailty based on TCM is currently unavailable. Consequently, the JFS, developed based on the aging principles of Kampo medicine, holds promise as a relevant and applicable tool for evaluating the Chinese population. Nevertheless, the absence of a validated Chinese version of the JFS restricts its utilization in primary care settings to assess frailty among Chinese-speaking individuals. Therefore, it is crucial to establish a validated Chinese version of the JFS to comprehensively assess frailty among the Chinese population and enhance the effectiveness of intervention strategies in mitigating adverse health outcomes among older adults. This study aims to undertake the translation and validation process for a simplified Chinese version of the JFS and subsequently evaluate its reliability and validity within older adults in China.

## 2. Materials and methods

### 2.1. Participants and data collection

This is a cross-sectional research study and employs the simple random sampling method. The recruitment of individuals aged 65 years and above was scheduled to take place from January 2023 to May 2023 at the Department of Preventive Care of Xi’an Honghui Hospital. Prior to their enrollment in the study, all participants provided written informed consent in accordance with the ethical guidelines outlined in the Helsinki Declaration (20). The inclusion criteria encompass individuals with the ability for reading and communication, independent mobility, and the capability to provide written informed consent. Exclusion criteria include diagnoses of psychiatric disorders, difficulties in understanding, as well as individuals who voluntarily decline to participate. During the recruitment period, a total of 208 individuals were deemed eligible. Among them, 32 were excluded due to their absence during participant assessment. There were no non-responders. Additionally, 16 individuals declined to participate, leaving a final sample size of 160 individuals. Ethical approval for this study was obtained from the Ethics Committee of Xi’an Honghui Hospital, with the assigned ethics approval number: 202305010.

All participants completed the assessment scales under the guidance of the research team members. Participants provided their personal information, such as age and gender, education, living situation. Furthermore, the most prevalent chronic diseases among elderly Chinese individuals were documented, primarily including hypertension, hyperlipidemia, diabetes, malignant neoplasms, stroke, chronic cardiac ailments, hepatic disorders, renal diseases, digestive disorders, pulmonary conditions, and arthritis (21). They also completed a set of patient-reported outcome (PRO) questionnaires, including JFS-C, SF-36, the Frail scale, and Barthel Index. All questionnaires were administered by the same person. Subsequently, a random sample of 60 participants was selected to complete the same JFS scale again after a period of 7–10 days from the initial administration.

### 2.2. Sample size

Sample size estimation was conducted using PASS 15 software for various analyses in this study. The test–retest reliability analysis performed on the study sample met the predetermined sample size requirements. The null hypothesis intraclass correlation coefficient (ICC) was set at 0.7, with the alternative hypothesis intraclass correlation coefficient set at 0.90. With a significance level of 0.05 and a power of 0.9, a minimum sample size of 25 individuals was determined to be adequate for this analysis (22). In terms of the internal consistency analysis, the sample size was determined based on the null hypothesis coefficient alpha of 0.7 and the actual coefficient alpha of 0.8, with a power of 0.9 and an alpha level of 0.05. Therefore, a minimum sample size of 131 individuals was required to achieve sufficient statistical power (23). Additionally, for the receiver operating characteristic (ROC) curve analysis, which necessitated an Area Under Curve (AUC) of 0.7 and a confidence level of 0.95, a minimum of 106 individuals was required (24).

### 2.3. Translation and cross-cultural adaptation procedure

A translation and back-translation approach were used to culturally adapt the JFS (25). Initially, the JFS was independently translated from English to Simplified Chinese by an orthopedic surgeon proficient in English and a senior English major, both native Chinese speakers with no medical background. The questionnaire items and scoring instructions were faithfully maintained in the same manner as the original English version, without any modifications. An experienced cross-cultural adaptation expert collaborated with the translators to merge the two translation versions into a unified version. Subsequently, two additional English-speaking individuals with no medical background independently back-translated the preliminary unified version into English. Through a rigorous comparison between the back-translated version and the original version, a revised Chinese version of the JFS was developed, employing a reconciliation process. To ensure the quality and validity of the Chinese version of the Japan Frailty Scale (JFS-C), a panel of 20 older individuals was invited to participate in preliminary testing of the pre-final version. This panel did not encounter any comprehension difficulties with the response options and did not provide substantial feedback or insights. Comprehensive discussions were held among all participating researchers, leading to the development of the final version of the JFS-C.

### 2.4. Instruments

#### 2.4.1. The Japan Frailty Scale

JFS was developed in 2022 by Japanese researchers (15). It comprises five items: nocturia (0–2 points), low back pain (0–2 points), cold hypersensitivity (0–2 points), exhaustion (0–4 points), and age (0–1 point). The cumulative score on the JFS ranges from 0 to 11 points. This tool is designed to be applied in primary care settings and can assist in early identification of pre-frail/frail individuals.

#### 2.4.2. The Frail scale

The Frail scale is a concise self-report instrument employed for frailty assessment (26). It serves as a valuable screening tool utilized by both healthcare professionals and non-professionals to identify individuals experiencing frailty. It includes items related to fatigue, resistance, walking, illness, and weight loss. The overall score of the scale ranges from 0 to 5, with a score of 0 denoting robustness, a score of 1 indicating pre-frailty, and a score of 2 or higher indicating the presence of frailty. The validity and reliability of the Chinese version of the Frail scale have been established, rendering it a suitable tool for evaluating frailty among older adults residing in the community setting in China (5).

#### 2.4.3. The Barthel index

The Barthel Index is a widely utilized scale for the routine assessment of activities of daily living (ADL) among older adults (27). It comprises 10 items, generating a comprehensive score ranging from 0 to 100. Within this scale, two items (bathing and grooming) are scored as either 0 or 5, while six items (feeding, dressing, bowel control, bladder control, toileting, and stair climbing) are assigned scores of 0, 5, or 10. The remaining two items (transfer and mobility) are scored as 0, 5, 10, or 15. Higher total scores and increased scores on individual items of the Barthel Index indicate a greater level of independence in performing ADLs. The psychometric properties of the Barthel Index have been found to be excellent, and the Chinese version of the scale has been widely implemented within the older population (28).

#### 2.4.4. The 36-item short-form health survey (SF-36)

The SF-36 is widely recognized as an instrumental tool for evaluating health-related quality of life (HRQoL) across diverse populations (29). This comprehensive health measurement incorporates eight multi-item dimensions that aim to assess functional status, overall well-being, and health evaluation. These dimensions encompass physical functioning (PF), role-physical (RP), bodily pain (BP), general health (GH), vitality (VT), social functioning (SF), role-emotional (RE), and mental health (MH). Each dimension focuses on specific aspects of an individual’s HRQoL, collectively providing a comprehensive assessment of their physical, emotional, and social well-being. Scores for each dimension are calculated by summing item scores and transforming them to a range from 0 (representing the poorest health status) to 100 (indicating the best health status). The SF-36 demonstrates excellent psychometric properties, including high internal consistency and good test–retest reliability (30). Moreover, it has demonstrated its applicability across diverse age groups and health conditions.

### 2.5. Statistical analysis

Continuous variables were summarized as mean ± standard deviation (SD), while categorical variables were presented as counts and percentages. Differences in JFS-C scores among subjects with different characteristics were assessed using T-tests and one-way analysis of variance (ANOVA) with Bonferroni correction. The relative reliability was evaluated through intra-class correlation coefficients (ICC), with ICC values indicating moderate (0.5–0.75), good (0.75–0.9), or excellent (>0.9) reliability (31). Internal consistency of the JFS-C scores was examined using Cronbach’s alpha coefficient, with values >0.70 considered adequate internal consistency. Absolute reliability was evaluated by calculating the standard error of measurement (SEM) and smallest detectable change (SDC). Pearson correlation coefficients (*r*) were used to investigate the association between JFS-C and the Frail scale, Barthel Index, and SF-36, with correlation strength interpreted as very strong (>0.80), strong (0.61–0.80), moderate (0.41–0.60), weak (0.21–0.40), or minimal to none (0.0–0.2) (32). Before this analysis, based on assessment of the content of the items on the scales, we hypothesized that the total scores of JFS-C correlated moderately with the total scores of the Barthel Index, the Frail scale, and SF-36. The validity of the standard and the optimal JFS-C cutoff point were determined using ROC analysis, based on the Youden index (sensitivity + specificity − 1). Statistical analyses were performed using MedCalc 20.0 and SPSS 26.0, with a significance level (α) set at 0.05.

## 3. Results

### 3.1. Participants and score distribution

A total of 160 individuals were enrolled in the study, predominantly female (58.1%, *n* = 93) and comprising 67 male participants, with a mean age of 82.9 years. A small proportion of participants (18.1%) had received education at the high school level or above. Approximately 24.4% of participants reported living alone. About 70% of participants had chronic diseases. The demographic and descriptive variables of the participants are shown in Table 1. In addition, Table 2 shows the JFS-C scores for different populations. Significantly disparate total scores on the JFS-C scale were observed based on gender, education, living situation, and the presence of chronic conditions. Specifically, women achieved significantly higher scores than men on the JFS-C. Furthermore, individuals living alone obtained significantly higher scores compared to those living with their family. Participants with lower education levels attained significantly higher scores compared to those with higher education levels. Lastly, participants with chronic diseases, especially those with a greater number of chronic conditions, scored significantly higher compared to those without chronic diseases.

**Table 1:** Socio-demographic and clinical variables of the participants.

Variables		(*n* = 160)
Age, mean ± SD		82.9 ± 10.2
Gender, *n* (%)	Male	67 (41.9)
	Female	93 (58.1)
Education, *n* (%)	Primary school or below	89 (55.6)
	Middle school	42 (26.3)
	High school or above	29 (18.1)
Living situation, *n* (%)	Living alone	39 (24.4)
	Living with family	121 (75.6)
Chronic diseases, *n* (%)	No chronic diseases	48 (30.0)
	One chronic disease	84 (52.5)
	Two chronic diseases	17 (10.6)
	Three or more chronic diseases	11 (6.9)
JFS-C, mean ± SD		5.4 ± 2.9
The Frail scale, mean ± SD		2.3 ± 1.4
Barthel Index, mean ± SD		76.5 ± 25
SF-36, mean ± SD	Physical Functioning (PF)	45.2 ± 39.5
	Role-Physical (RP)	64.1 ± 43.5
	Bodily Pain (BP)	69.9 ± 32.1
	General Health (GH)	52.9 ± 26.9
	Vitality (VT)	59.4 ± 23.7
	Social Functioning (SF)	55.8 ± 33.1
	Health Transition (HT)	33.6 ± 21.5
	Mental Health (MH)	66.8 ± 21.9
	Role-Emotional (RE)	77.9 ± 36.2

SD indicates standard deviation. The scores of the JFS-C range from 0 to 11. The scores of the Frail scale range from 0 to 5. The scores of the Barthel Index range from 0 to 100. The scores of the SF-36 subscales range from 0 to 100.

**Table 2:** JFS-C scores in different populations.

Characteristics		Total score of JFS-C	*p* value
		(Mean ± SD)	
Gender	Male	4.7 ± 2.6^a^	0.007
	Female	6.0 ± 3.0^a^	
Education	Primary school or below	6.3 ± 3.0^a^	<0.001
	Middle school	5.0 ± 2.3	
	High school or above	3.2 ± 2.2^a^	
Living situation	Living alone	7.0 ± 3.1^a^	<0.001
	Living with family	4.9 ± 2.7^a^	
Chronic diseases	No chronic diseases	3.8 ± 2.0^a^	<0.001
	One chronic disease	5.0 ± 2.5^a^	
	Two chronic diseases	8.8 ± 1.7^a^	
	Three or more chronic diseases	10.5 ± 0.5^a^	

SD, standard deviation; ^a^item with significant difference.

### 3.2. Reliability

The Cronbach’s alpha coefficient for JFS-C was calculated to be 0.711. The test–retest analysis involving 60 patients revealed a high ICC of 0.949 (95% CI 0.916–0.969). The SEM, which reflects the systematic and random errors of an instrument not attributed to true changes, was 0.663. The SDC, representing the smallest change in score that presumably reflects the true change above measurement error, was 1.838.

### 3.3. Convergent validity

Pearson correlation analysis demonstrated a significant and strong positive correlation (*r* = 0.786, *p* < 0.001) between JFS-C and the Frail scale. JFS-C also exhibited a moderate negative correlation with the Barthel Index (*r* = −0.598, *p* < 0.001). When compared to the SF-36, JFS-C demonstrated significant negative correlations with the PF (*r* = −0.598, *p* < 0.001), RP (*r* = −0.581, *p* < 0.001), BP (*r* = −0.574, *p* < 0.001), GH (*r* = −0.636, *p* < 0.001), VT (*r* = −0.653, *p* < 0.001), SF (*r* = −0.657, *p* < 0.001), and HT (*r* = −0.661, *p* < 0.001) subscales. However, weak correlations were found between JFS-C and the MH (*r* = −0.363, *p* < 0.001) and RE (*r* = −0.350, *p* < 0.001) subscales of the SF-36. These findings support the convergent or discriminant validity of JFS-C in frail patients in China (Table 3).

**Table 3:** Convergent validity of JFS-C.

Scales		Pearson correlation (*r*)
The Frail scale		0.786^*^
Barthel Index		−0.598^*^
SF-36	Physical Functioning (PF)	−0.598^*^
	Role-Physical (RP)	−0.581^*^
	Bodily Pain (BP)	−0.574^*^
	General Health (GH)	−0.636^*^
	Vitality (VT)	−0.653^*^
	Social Functioning (SF)	−0.657^*^
	Health Transition (HT)	−0.661^*^
	Mental Health (MH)	−0.363^*^
	Role-Emotional (RE)	−0.350^*^

*Correlation is significant at the 0.01 level (2-tailed).

### 3.4. Criterion validity

**Figure 1:**
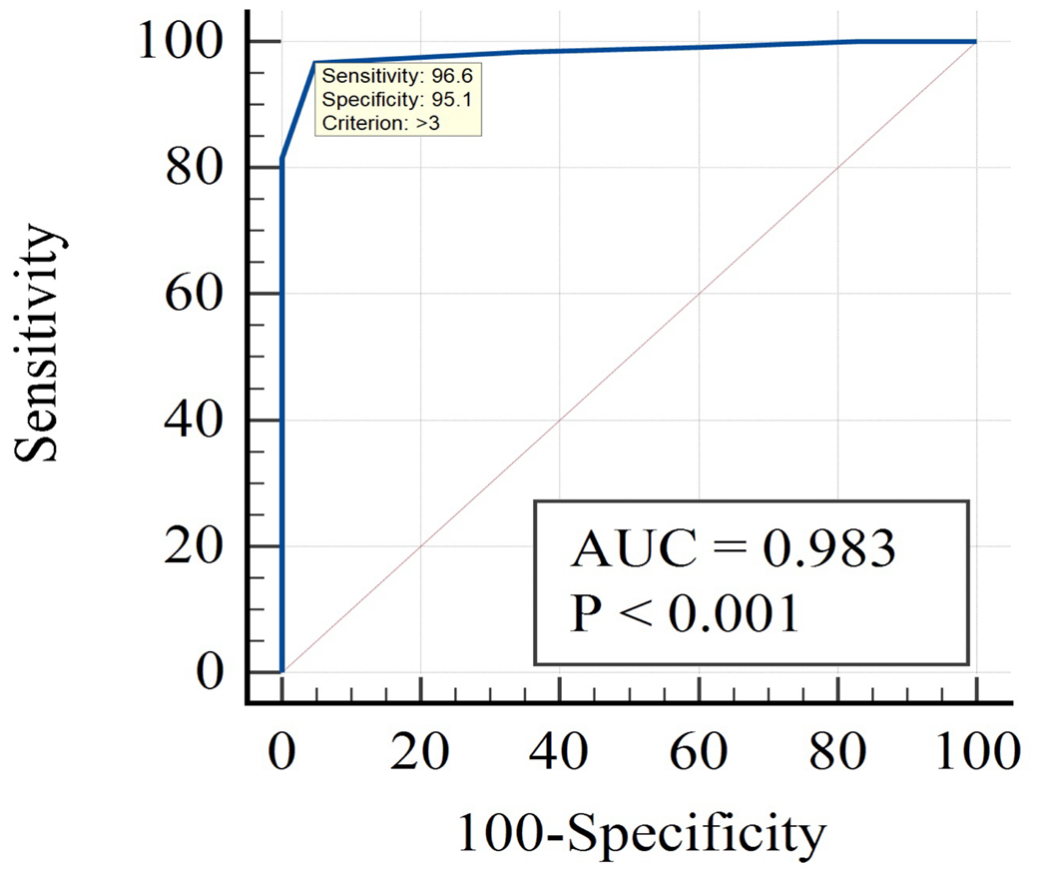
The ROC curve analysis of JFS-C using the Frail scale phenotype as a standard.

Figure 1 presents the results of the ROC analysis using the Frail scale as the reference standard, with a cutoff value of ≥2 on the Frail scale indicating frailty. When employing the Frail scale as the standard, the prevalence of frailty was found to be 74.4%. The results showed that the AUC for the JFS-C was 0.983 (95% confidence interval: 0.949–0.997). Based on the maximum Youden index, the optimal cutoff point for the JFS-C in the Chinese population was determined to be 3, with a sensitivity of 96.6% and specificity of 95.1%. The positive predictive value (PPV) was 98.29% and the negative predictive value (NPV) was 90.70%. Based on the cutoff value, the JFS-C successfully identified frailty in 73.1% of cases.

## 4. Discussion

In the present study, our objective was to extend the application of the JFS beyond its original Japanese population and examine its efficacy in quantifying frailty levels among older individuals in China. This research fills a crucial gap by introducing a standardized tool for assessing frailty in a culturally diverse context. The findings of our study demonstrate the potential of the JFS as a valuable measure for evaluating frailty in Chinese older individuals.

The present study yielded promising results regarding the internal consistency and test–retest reliability of the JFS-C in assessing frailty among the Chinese older population. The calculated Cronbach’s alpha coefficient of 0.711 indicated adequate internal consistency, suggesting adequate coherence among the items of the JFS-C. Moreover, the excellent test–retest reliability, as reflected by the ICC of 0.949, demonstrated the stability and consistency of the JFS-C over time. These findings provide further evidence supporting the validity and reliability of the JFS-C as a robust tool for assessing frailty in the Chinese older population.

One important aspect of this study is the cultural adaptation of the JFS for the Chinese population. Despite cultural differences, participants in our study willingly participated and were able to complete the JFS assessment independently. This highlights the acceptability and feasibility of using the JFS-C in Chinese older individuals. The similarity between Kampo medicine, on which the JFS is based, and traditional Chinese medicine likely contributed to the ease of understanding and completion of the questionnaire. Thus, the JFS-C achieved semantic, idiomatic, and experiential equivalence in assessing frailty among the older population in China.

To assess the construct validity of the JFS-C, we examined its correlations with assessment of frailty (the Frail scale), ADL(Barthel Index), and HRQoL(SF-36). The original version of the questionnaire study showed a moderate correlation between JFS and the Kihon Checklist and Locomo-5 (15), which is a questionnaire measuring elderly mobility function The Kihon Checklist is a questionnaire that reflects frailty status (33). Locomo-5 is a questionnaire that reflects elderly physical function, which is similar to our research design. Our results indicated good correlations between the JFS-C and these measures. Specifically, the JFS-C exhibited strong correlations with the Frail scale (*r* = 0.786, *p* < 0.001), moderate correlations with the Barthel Index (*r* = −0.598, *p* < 0.001), and moderate correlations with disease-related domains of the SF-36 (*r* = −0.574 to −0.661), indicating its ability to capture frailty-related dimensions. Weaker correlations were observed in the SF-36 MH(*r* = −0.363, *p* < 0.001) domain and RE domain (*r* = −0.350, *p* < 0.001), consistent with previous research emphasizing the stronger association of frailty with physical aspects (34, 35). These results further support the validity of the JFS-C in capturing the frailty within the Chinese older population.

In terms of criterion validity, the JFS-C demonstrated excellent discriminatory ability in identifying frailty when compared to the Chinese FRAIL scale, which served as the reference standard. The AUC for the JFS-C was 0.983, indicating high discriminative power. Compared to the original study’s cutoff value of 3/4, at the optimal cutoff point of 3, the JFS-C demonstrated a sensitivity of 96.6% and a specificity of 95.1%, surpassing the findings of the original study (sensitivity: 80.4%, specificity: 71.3%) (15). The PPV for JFS-C was determined to be 98.29%, and the NPV was 90.70%. These values were both higher than those reported in the original study, which had a PPV of 69.3% and an NPV of 73.7% (15). This discrepancy can be attributed to the higher prevalence of frailty, which tends to result in an elevated PPV but a lower NPV (36). These findings highlight that JFS-C serves as an ideal screening tool for frailty, given its higher accuracy in identifying frail individuals (98.29%).

There are significant differences in JFS-C total scores among individuals with different demographic characteristics. Specifically, females tend to have significantly higher JFS scores compared to males, consistent with previous research indicating that female participants recognize more vulnerable individuals than their peers (5). Additionally, individuals with lower educational attainment, those living independently, and those with a higher number of chronic disease types also demonstrated elevated JFS scores. This is because socio-demographic status and dependency are risk factors leading to frailty (37–39). Therefore, healthcare professionals can utilize JFS-C to identify these specific groups and implement proactive measures, such as promoting healthy dietary habits and encouraging regular physical exercise, for the prevention of frailty in non-frail populations (40–42). For frail individuals, proactive identification and multidisciplinary interventions should be implemented (43, 44).

There are certain limitations to our study that should be acknowledged. Firstly, we employed the Frail scale as the external criterion for JFS-C; however, it should be noted that the Frail scale itself does not represent an absolute standard for measuring frailty. Therefore, future longitudinal studies are warranted to further validate the accuracy of JFS. Secondly, since our study participants were exclusively from a single city in China, caution should be exercised when attempting to generalize the findings to broader populations.

## 5. Conclusion

The current study has demonstrated the reliability and validity of the JFS-C as a valuable tool for assessing frailty in the older population of China. With the utilization of JFS-C, clinical practitioners specializing in traditional Chinese medicine can enhance their ability to assess frailty status among the elderly in China, thus elevating the quality of healthcare services for this particular demographic in both community and hospital settings.

## Data availability statement

The original contributions presented in the study are included in the article/supplementary material, further inquiries can be directed to the corresponding authors.

## Ethics statement

The studies involving humans were approved by the Ethics Committee of Xi'an Honghui Hospital. The studies were conducted in accordance with the local legislation and institutional requirements. The participants provided their written informed consent to participate in this study.

## Author contributions

DPW: Conceptualization, Data curation, Investigation, Software, Writing – original draft. RW: Data curation, Formal analysis, Investigation, Writing – original draft. JW: Conceptualization, Data curation, Project administration, Software, Writing – original draft. QZ: Conceptualization, Methodology, Validation, Writing – review & editing. LS: Conceptualization, Funding acquisition, Resources, Supervision, Validation, Writing – review & editing. JBM: Data curation, Validation, Writing – review & editing. SXY: Conceptualization, Methodology, Project administration, Validation, Visualization, Writing – review & editing. CX: Writing – review & editing.

## Funding

The author(s) declare financial support was received for the research, authorship, and/or publication of this article. This work was supported by Key Research and Development Program of Shaanxi Province (Grant no. 2023-YBSF-464); General Research Plan of Xi’an Health Commission (Grant no. 2023yb30); the National Natural Science Foundation of China (Grant no. 82173627).

## Conflict of interest

The authors declare that the research was conducted in the absence of any commercial or financial relationships that could be construed as a potential conflict of interest.

## Publisher’s note

All claims expressed in this article are solely those of the authors and do not necessarily represent those of their affiliated organizations, or those of the publisher, the editors and the reviewers. Any product that may be evaluated in this article, or claim that may be made by its manufacturer, is not guaranteed or endorsed by the publisher.

## Abbreviations

JFS: Japan Frailty Scale
TCM: Traditional Chinese Medicine
ICC: intraclass correlation coefficient
AUC: Area Under Curve
PRO: patient-reported outcome
ROC: receiver operating characteristic
SEM: standard error of measurement
SDC: smallest detectable change
PPV: positive predictive value
NPV: negative predictive value
